# Spectrum of Pediatric Surgical Cases at a Private Pediatric Specialized Hospital in Bangladesh: A Retrospective Study

**DOI:** 10.7759/cureus.106236

**Published:** 2026-03-31

**Authors:** Fatima T Jannat, Shakhawat Islam, Md Nazrul Islam, Mohammad Nooruzzaman, Pravin Chaudhary, Md Ruhul Amin

**Affiliations:** 1 Department of Pediatric Surgery, Nobojatok-Shishu and General Hospital, Dhaka, BGD; 2 Department of Pediatric Surgery, Sir Salimullah Medical College Mitford Hospital, Dhaka, BGD; 3 Department of Pediatric Surgery, Bangladesh Medical University, Dhaka, BGD; 4 Department of Pediatric Surgery, Khulna Medical University, Khulna, BGD

**Keywords:** congenital anomalies, genitourinary anomalies, pediatric surgery, pediatric surgical workload, surgical case spectrum

## Abstract

Background

Pediatric surgical conditions represent an important component of child health services in low- and middle-income countries (LMICs). Understanding the spectrum of surgical conditions presenting to hospitals is essential for service planning, resource allocation, and improving surgical outcomes. However, data describing patterns of pediatric surgical cases in specialized hospitals in Bangladesh remain limited.

Objective

This study aimed to describe the spectrum of pediatric surgical cases managed at a private pediatric specialized hospital in Bangladesh among children aged 0-17 years, including perioperative outcomes.

Methods

This retrospective study reviewed all pediatric surgical cases managed at Nobojatok-Shishu and General Hospital, Dhaka, Bangladesh, between May 2023 and April 2024. Data collected included age, sex, diagnosis, surgical procedures performed, and perioperative mortality outcomes. Diagnoses were categorized into major diagnostic groups: genitourinary, gastrointestinal, nervous system, neoplasms, and injury/trauma. Descriptive statistics were used to summarize frequencies and proportions.

Results

A total of 265 pediatric surgical cases were included. The mean age was 4.8 ± 4.8 years, with children aged one to five years constituting the largest group (109/265; 41.1%). There was a male predominance (198/265; 74.7%). Genitourinary conditions accounted for the largest proportion of cases (163/265; 61.5%), followed by gastrointestinal disorders (68/265; 25.7%) and neoplasms (15/265; 5.7%). The most frequent diagnoses were inguinal hernia (33/265; 12.5%), hypospadias (31/265; 11.7%), and undescended testis (24/265; 9.1%). The most commonly performed procedures were urethroplasty (34/265; 12.8%), herniotomy (32/265; 12.1%), and orchiopexy (25/265; 9.4%). Three perioperative deaths occurred during the study period (3/265; 1.1%), all among male patients.

Conclusions

Genitourinary anomalies and gastrointestinal surgical conditions constitute a major proportion of the pediatric surgical workload in this hospital. These findings highlight the need for strengthened pediatric surgical services within similar specialized settings in Bangladesh.

## Introduction

Pediatric surgery is an essential but often under-prioritized component of child health services in low- and middle-income countries (LMICs) [1]. Globally, surgical conditions contribute significantly to morbidity and mortality among children, with congenital anomalies, infections, trauma, and neoplastic conditions forming a substantial portion of the surgical burden [2,3]. Despite increasing recognition of global surgery as a public health priority, pediatric surgical services remain unevenly distributed, particularly in resource-limited settings [1,3].

In many LMICs, pediatric surgical care is concentrated in tertiary government hospitals, with limited data available from private or specialized centers. However, private pediatric specialized hospitals are increasingly playing an important role in delivering surgical services, particularly in urban settings where access to specialized care is expanding [4,5]. Understanding the pattern of surgical diseases in such settings is important for planning workforce development, infrastructure investment, and training priorities.

Previous studies from Africa and other LMICs have demonstrated that congenital anomalies, genitourinary conditions, and digestive system disorders constitute a large proportion of pediatric surgical workload. Studies evaluating pediatric surgical admissions have reported that inguinal hernia repair, appendicitis, and congenital malformations are among the most common indications for surgery [6,7]. Institutional audits are important for improving the quality of care and guiding service expansion.

Bangladesh, like many LMICs, faces a dual burden of congenital and acquired surgical conditions among children [8,9]. Rapid urbanization, improved healthcare access, and increasing awareness among caregivers have contributed to increased utilization of specialized pediatric surgical services [10,11]. However, there remains limited published evidence describing the spectrum of pediatric surgical cases managed in private specialized hospitals within the country.

An appraisal of surgical patterns within individual institutions provides valuable insights into disease distribution, age and sex patterns, procedural demand, and outcomes. Such information is essential for rational allocation of resources, development of subspecialty services, and strengthening referral pathways. Furthermore, documenting surgical case patterns contributes to the broader understanding of pediatric surgical epidemiology in South Asia.

This study, therefore, aimed to describe the spectrum of pediatric surgical cases managed at a private pediatric specialized hospital in Bangladesh over a one-year period. Specifically, the study examined demographic characteristics, diagnostic categories, types of surgical procedures performed, and perioperative mortality outcomes among pediatric patients undergoing surgery.

## Materials and methods

Study design and setting

This was a retrospective descriptive study conducted at Nobojatok-Shishu and General Hospital, Dhaka, Bangladesh. The hospital serves as a referral center for pediatric surgical cases from urban and peri-urban areas and provides both elective and emergency surgical services for children.

Study period and population

All pediatric patients who underwent surgical procedures between May 2023 and April 2024 were included in the study. Children aged from birth to 17 years who underwent surgical intervention during the study period were eligible for inclusion.

Inclusion and exclusion criteria

All pediatric patients who underwent operative procedures by the pediatric surgical unit during the study period were included. Patients managed conservatively without surgery and minor outpatient procedures not requiring operative intervention were excluded.

Data collection

Data were retrieved from hospital surgical records and operative registers using a standardized data extraction format capturing demographic, clinical, and procedural variables. Age groups were defined as: <28 days, 28 days to <1 year, 1-5 years, 6-10 years, 11-14 years, and >14 years. Variables collected included age at surgery, sex, diagnosis, surgical procedure performed, and perioperative outcome, including mortality. Diagnoses were categorized into six major groups: genitourinary, gastrointestinal, nervous system, neoplasms, injury/trauma, and miscellaneous conditions.

Outcome measures

The primary outcome was the distribution of pediatric surgical diagnoses and procedures. Secondary outcomes included age and sex distribution and perioperative mortality.

Statistical analysis

Data were entered and analyzed using SPSS version 26 (IBM Inc., Armonk, New York). Continuous variables were summarized using means and standard deviations, or median and interquartile range (IQR), while categorical variables were summarized using frequencies and percentages. Results were presented using tables and figures, including distribution charts and box-and-whisker plots to demonstrate age distribution across diagnostic categories.

## Results

Socio-demographic characteristics

**Figure 1:**
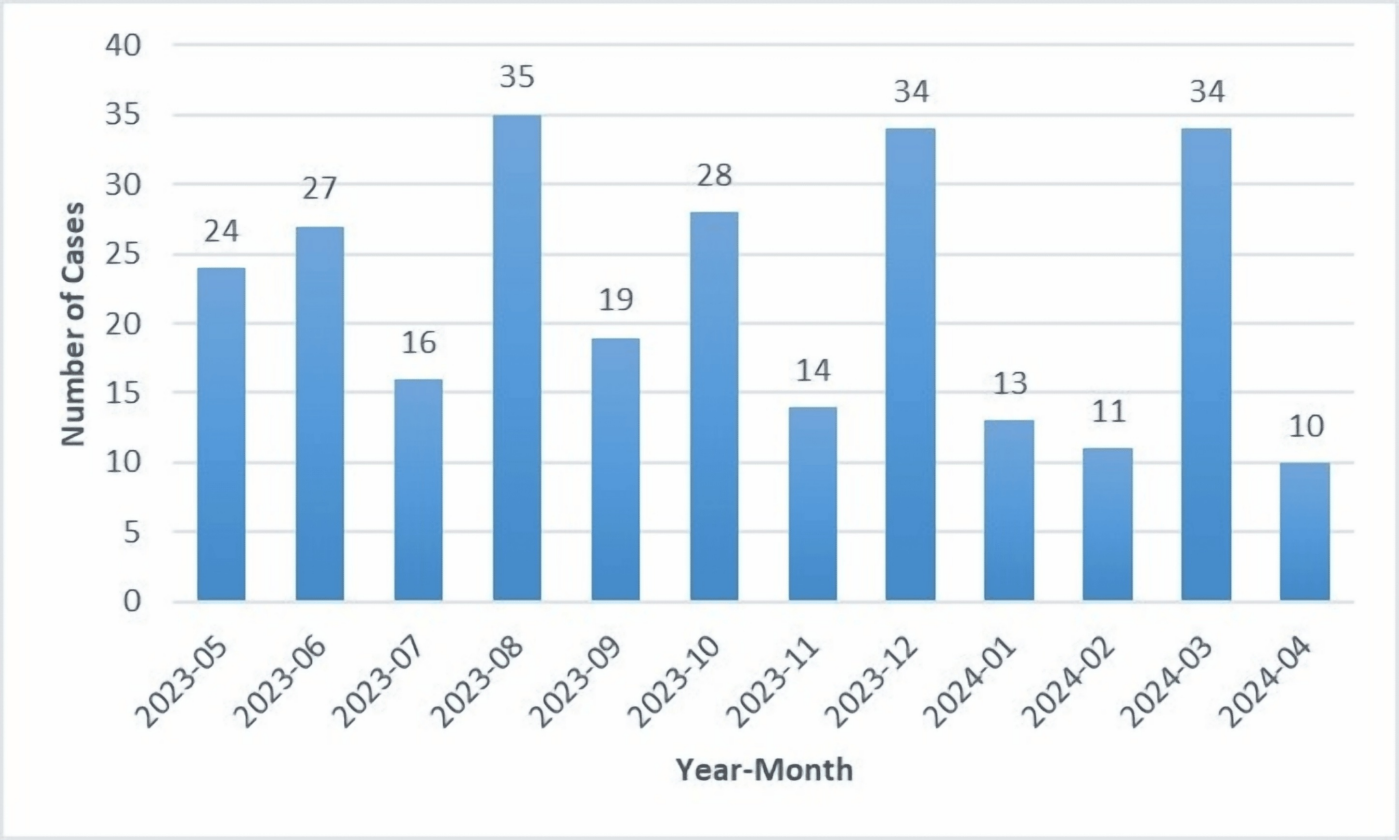
Monthly distribution of pediatric surgical cases from May 2023 to April 2024

Table 1 shows that 265 pediatric surgical cases were analyzed during the study period. The mean age of the patients was 4.8 ± 4.8 years. Children aged between one and five years constituted the largest proportion of cases (109/265; 41.1%). The male-to-female ratio was approximately 3:1. The monthly distribution of surgical cases showed relatively consistent case volumes throughout the study period (Figure 1).

**Table 1:** Socio-demographic characteristics of the study population

Socio-demographic characteristics	Frequency (%)
Age	
<28 days	16 (6.0)
28 days to <1 year	49 (18.5)
1–5 years	109 (41.1)
6–10 years	51 (19.2)
11–14 years	22 (8.3)
>14 years	17 (6.4)
Mean ± SD (years)	4.8 ± 4.8
Minimum - Maximum (years)	0.0 - 17.0
Sex	
Male	198 (74.7)
Female	67 (25.3)
Total	265 (100.0)

**Figure 2:**
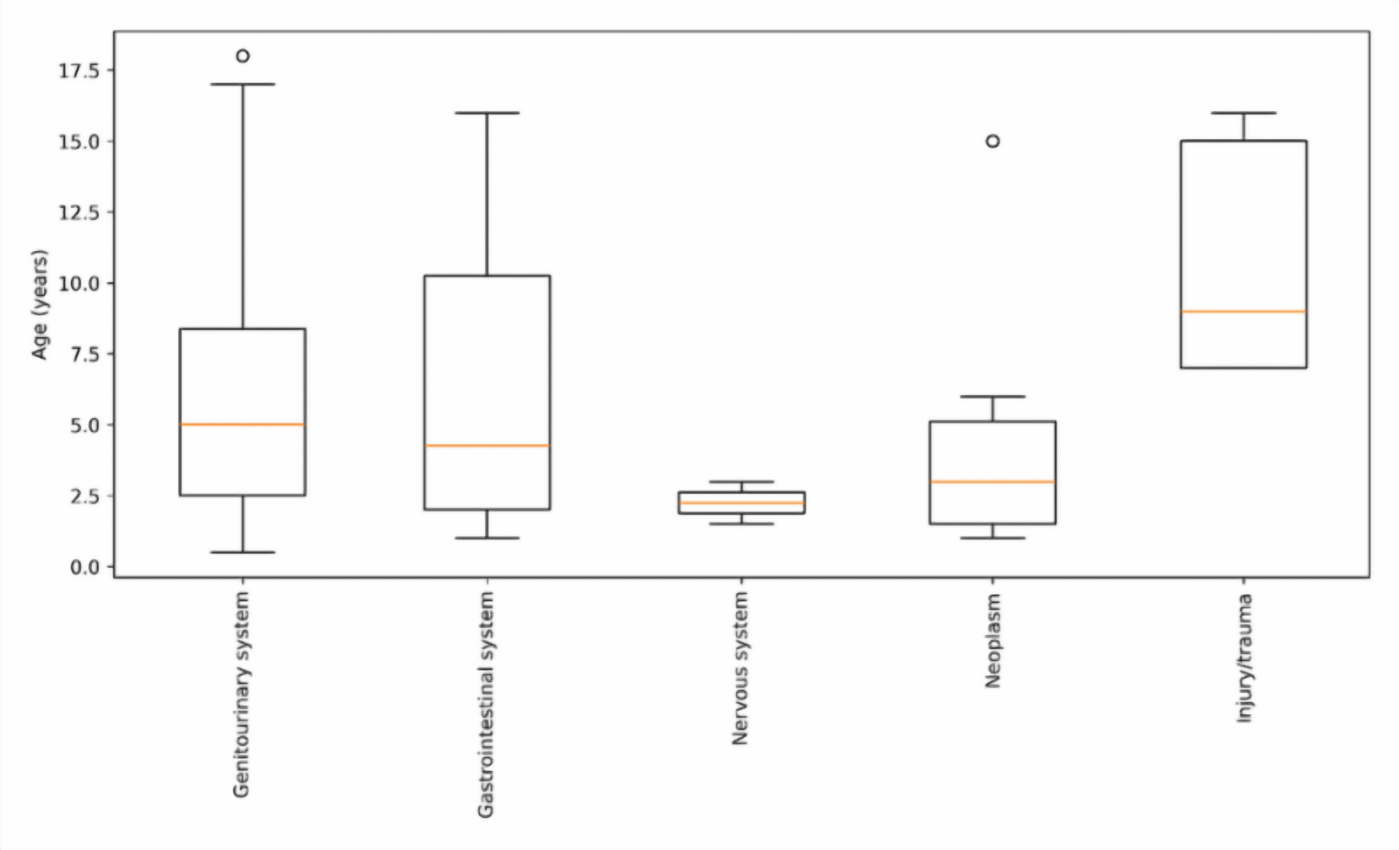
Boxplot showing the age distribution of pediatric surgical patients across diagnostic categories (genitourinary, gastrointestinal, nervous system, neoplasm, and injury/trauma) Boxes represent the interquartile range with median values indicated by the central line, and whiskers represent the range of observed values.

Figure 2 shows the age distribution of pediatric surgical patients across different diagnostic systems. Children with injury/trauma had the highest median age (around nine years), followed by genitourinary and gastrointestinal conditions with median ages around five and four years, respectively. Nervous system disorders occurred predominantly in younger children (median ≈2 years) with a narrow age range, while neoplasm cases showed moderate age variability. Overall, the figure indicates that most congenital conditions were diagnosed at younger ages, whereas trauma-related cases occurred in relatively older children.

Pattern of pediatric surgical diagnoses

Table 2 presents the distribution of pediatric surgical diagnoses by organ system. Genitourinary conditions were the most common, accounting for 163/265 cases (61.5%). The leading diagnoses were inguinal hernia (33/265; 12.5%), hypospadias (31/265; 11.7%), and undescended testis (24/265; 9.1%). Gastrointestinal disorders accounted for 68/265 cases (25.7%), most frequently Hirschsprung disease (12/265; 4.5%) and anorectal malformation (8/265; 3.0%). Less frequent categories included neoplasms (15/265; 5.7%), injury/trauma (9/265; 3.4%), and nervous system disorders (5/265; 1.9%). Overall, the majority of patients were male (198/265, 74.7%), reflecting the predominance of male-specific genitourinary conditions in the study population.

**Table 2:** Spectrum of pediatric surgical diagnoses by organ system † Other genitourinary anomalies include hydronephrosis variants, vesicoureteral reflux variants, neurogenic bladder, meatal stenosis, vaginal atresia, and other rare congenital or postoperative genitourinary conditions. * Other gastrointestinal anomalies include rare conditions such as intestinal atresia, malrotation of the gut, neonatal intestinal obstruction, tracheoesophageal fistula, and other uncommon congenital gastrointestinal disorders. ‡ Miscellaneous conditions include diagnoses not clearly classified within major systems, such as isolated soft-tissue lesions, minor congenital anomalies, and other infrequently encountered surgical conditions. Percentages were calculated using the total sample size (N=265). Rare diagnoses with fewer than three cases were grouped within system-specific "other" categories for clarity. PUJ - pelviureteric junction

System / diagnosis	n (%)	Male	Female
Genitourinary system (n=163)			
Inguinal hernia	33 (12.5)	29	4
Hypospadias	31 (11.7)	31	0
Undescended testis	24 (9.1)	24	0
Exstrophy–epispadias complex	15 (5.7)	12	3
Hydronephrosis / PUJ obstruction	9 (3.4)	8	1
Posterior urethral valve	5 (1.9)	5	0
Disorders of sex development	5 (1.9)	4	1
Hydrocele	4 (1.5)	4	0
Epispadias	4 (1.5)	4	0
Urethrocutaneous fistula	3 (1.1)	3	0
Vesicoureteral reflux	3 (1.1)	2	1
Other genitourinary anomalies†	27 (10.2)	17	10
Gastrointestinal system (n=68)			
Hirschsprung disease	12 (4.5)	7	5
Anorectal malformation	8 (3.0)	4	4
Chronic constipation	7 (2.6)	5	2
Acute appendicitis	4 (1.5)	2	2
Cholelithiasis	4 (1.5)	3	1
Cleft palate / cleft lip	4 (1.5)	2	2
Perianal fistula	3 (1.1)	2	1
Umbilical hernia	3 (1.1)	2	1
Other gastrointestinal anomalies*	23 (8.7)	20	3
Nervous system (n=5)			
Meningomyelocele	3 (1.1)	0	3
Hydrocephalus	2 (0.8)	1	1
Neoplasms (n=15)			
Lipoma	4 (1.5)	0	4
Wilms tumor	4 (1.5)	2	2
Dermoid cyst	3 (1.1)	1	2
Neuroblastoma	2 (0.8)	1	1
Sacrococcygeal teratoma	1 (0.4)	1	0
Yolk sac tumor (testis)	1 (0.4)	1	0
Injury / trauma (n=9)			
Enterocutaneous fistula / trauma-related cases	7 (2.6)	3	4
Abdominal injury	2 (0.8)	2	0
Miscellaneous conditions‡ (n=5)	5 (1.9)	4	1
Total	265 (100)	198	67

Surgical procedures performed

The ten most frequently performed surgical procedures accounted for 138/265 operations (52.1%). Urethroplasty was the most common procedure (34/265; 12.8%), followed by herniotomy (32/265; 12.1%) and orchiopexy (25/265; 9.4%). Ritual circumcision was performed in 13/265 cases (4.9%). Other commonly performed procedures included transanal pull-through for Hirschsprung disease (11/265; 4.2%), repair of exstrophy-epispadias complex (6/265; 2.3%), colostomy closure (5/265; 1.9%), appendicectomy (5/265; 1.9%), and posterior sagittal anorectoplasty or anterior sagittal anorectoplasty (5/265; 1.9%) (Table 3).

**Table 3:** Top ten pediatric surgical procedures performed Percentages were calculated using the total sample size (N=265) PSARP - posterior sagittal anorectoplasty; ASARP - anterior sagittal anorectoplasty

Rank	Procedure performed	Frequency (%)
1	Urethroplasty	34 (12.8)
2	Herniotomy	32 (12.1)
3	Orchiopexy	25 (9.4)
4	Ritual circumcision	13 (4.9)
5	Transanal pull-through	11 (4.2)
6	Repair of EEC	6 (2.3)
7	Colostomy closure	5 (1.9)
8	Bladder neck reconstruction	3 (1.1)
9	Appendicectomy	5 (1.9)
10	PSARP / ASARP	5 (1.9)
	Total (top 10 procedures)	138 (52.1)

Perioperative mortality outcomes

Three deaths were recorded at the time of surgery during the study period, yielding an overall mortality rate of 1.1%. All deaths occurred in male patients. One death was associated with splenic injury in a seven-year-old child, while two deaths occurred among neonates with tracheo-esophageal fistula (Table 4).

**Table 4:** Diagnosis, age, and sex distribution of mortality cases during surgery

Case No.	Diagnosis	Age	Sex
1	Splenic injury	7 years	Male
2	Tracheo-esophageal fistula	15 days	Male
3	Tracheo-esophageal fistula	4 days	Male

## Discussion

This study provides an overview of the spectrum of pediatric surgical cases managed in a private pediatric specialized hospital in Bangladesh and highlights important patterns in disease distribution and perioperative surgical outcomes. The findings demonstrate that genitourinary and gastrointestinal conditions form a substantial component of pediatric surgical workload in this setting.

The predominance of male patients observed in this study is consistent with reports from other LMICs [12,13]. This pattern may partly be explained by the high frequency of male-specific genitourinary conditions such as hypospadias and undescended testis [14], both of which represented major contributors to surgical workload in the present study. Similar findings have been reported in studies from Africa and Asia, where congenital urogenital anomalies account for a significant proportion of pediatric surgical procedures [15,16].

The age distribution in this study demonstrates that the majority of surgical cases occurred among children under five years of age. This observation is consistent with global data indicating that early childhood represents a critical period for diagnosis and surgical correction of congenital anomalies [17,18]. Early intervention is particularly important for conditions such as Hirschsprung disease, anorectal malformations, and hydronephrosis, where delayed treatment may lead to functional impairment and long-term complications [19].

Unlike several studies from other LMIC settings, where gastrointestinal system disorders dominate pediatric surgical workload [20], genitourinary conditions were the leading category in this study. Inguinal hernia repair has consistently been reported as one of the most frequent operations performed by pediatric surgeons worldwide [21]. This difference may reflect referral patterns and the specialized nature of the hospital, which likely receives a higher proportion of elective reconstructive urological cases. The availability of specialized pediatric surgical expertise may also influence case mix, with caregivers preferentially seeking care for complex congenital conditions in private specialized centers.

Gastrointestinal system disorders nevertheless represented a substantial proportion of surgical cases, with Hirschsprung disease and anorectal malformation being the most common conditions. This finding is consistent with previous literature [22,23]. The relatively lower proportion of appendicitis observed in this study compared to some African studies [6] may be attributable to differences in referral pathways.

Developmental anomalies constituted an important group of conditions requiring surgical management. The presence of complex anomalies such as exstrophy-epispadias complex underscores the need for multidisciplinary care, including pediatric urology, neonatology, and specialized anesthesia services [18]. The increasing recognition of congenital anomalies as a significant contributor to childhood morbidity in LMICs highlights the importance of strengthening early diagnosis and referral systems.

The proportion of trauma-related surgical cases in this study was relatively low compared to reports from tertiary trauma centers. This likely reflects institutional case selection, as orthopedic and neurosurgical trauma cases may be managed in other specialized units. Similar observations have been reported in institutional audits where case mix varies according to hospital specialization [24].

The overall perioperative mortality rate of 1.1% observed in this study is lower than rates reported in several LMIC settings, where mortality rates between 5% and 10% have been documented [6,12]. This relatively lower rate should be interpreted in the context of a higher proportion of elective procedures and the specialized nature of the study setting. However, this study assessed only immediate perioperative mortality and did not capture postoperative complications or long-term outcomes. The occurrence of mortality among neonates with tracheo-esophageal fistula highlights the ongoing challenges associated with neonatal surgery, particularly in resource-constrained environments where intensive care support may be limited.

This study provides novel insights into the spectrum of pediatric surgical conditions in a private specialized hospital in Bangladesh, an underreported setting. The comprehensive collection of data on demographics, diagnoses, procedures, and perioperative outcomes, along with comparisons to existing LMIC literature, enhances the relevance of the findings. These data offer useful evidence for service planning, workforce development, and resource allocation within similar specialized and urban healthcare settings, and may inform future multi-center research and policy discussions.

This study has several limitations. As a single-centre retrospective study, the findings may not be generalizable to other healthcare settings in Bangladesh, particularly public or rural facilities. The retrospective design is subject to potential inaccuracies or incomplete medical records. The relatively small sample size may also limit the representativeness of the observed case spectrum. The study also did not include postoperative complications or long-term outcomes, limiting the assessment of overall surgical outcomes. Furthermore, referral and selection bias inherent to a private specialized hospital may have influenced the observed disease spectrum. Therefore, the findings should be interpreted within the context of this specific institutional setting.

## Conclusions

Genitourinary and gastrointestinal surgical conditions constitute the major components of pediatric surgical workload in this private pediatric specialized hospital in Bangladesh. The predominance of congenital and elective surgical conditions reflects evolving patterns of pediatric surgical care in urban LMIC settings. Institutional audits such as this provide important facility-level insights for planning pediatric surgical services and guiding future research in Bangladesh, but broader generalization requires multi-center evidence.
